# A Shandon PapSpin liquid-based gynecological test: A split-sample and direct-to-vial test with histology follow-up study

**DOI:** 10.4103/1742-6413.61200

**Published:** 2010-03-20

**Authors:** J Rimiene, J Petronytė, Z Gudleviciene, Giedrė Smailytė, Ingrida Krasauskaite, A Laurinavicius

**Affiliations:** 1 National Centre of Pathology, Vilnius University, Vilnius, Lithuania; 2 Vilnius University Faculty of Medicine, Vilnius University, Vilnius, Lithuania; 3 Institute of Oncology, Vilnius University, Vilnius, Lithuania

**Keywords:** Cervical cancer, cervical intraepithelial neoplasia, liquid based cytology, Papanicolaou test

## Abstract

**Background:**

Studies for liquid-based Papanicolaou (Pap) tests reveal that liquid-based cytology (LBC) is a safe and effective alternative to the conventional Pap smear. Although there is research on ThinPrep and SurePath systems, information is lacking to evaluate the efficiency and effectiveness of systems based on cytocentrifugation. This study is designed to determine the sensitivity and specificity of the Shandon PapSpin (ThermoShandon, Pittsburgh, Pennsylvania, USA) liquid-based gynecological system. We used split-sample and direct-to-vial study design.

**Materials and Methods:**

2,945 women referred to prophylactic check-up were enrolled in this study. Split sample design was used in 1,500 women and residual cervical cytology specimen from all these cases was placed in fluid for PapSpin preparation after performing conventional smear. The direct-to-vial study was carried out in another cohort of 1,445 women in whom the entire cervical material was investigated using only the PapSpin technique. Follow up histological diagnoses for 141 women were obtained from both study arms following 189 abnormal cytology cases. 80 LBC cases from the split sample group and 61 LBC cases in the direct-to-vial group were correlated with the histology results. The sensitivity and secificity of the conventional smear and PapSpin tests in both study arms were compared.

**Results:**

In the split sample group, conventional smears showed a higher proportion of ASC-US (atypical cells undetermined significance): 31 (2.1%) *vs* 10 (0.7%) in PapSpin (*P* = 0.001). A higher proportion of unsatisfactory samples was found in the conventional smear group: 25 (1.7%) *vs* 6 (0.4%) cases (*P* = 0.001). In the split sample group, the sensitivity of the conventional and PapSpin tests was 68.7% *vs* 78.1%, and the specificity 93.8% *vs* 91.8%, respectively. In the direct to vial group PapSpin sensitivity was 75.9% and specificity 96.5%. The differences in sensitivity and specificity were not significant. The positive predictive values for the conventional and PapSpin methods were not different in the split sample group: 88.0% *vs* 86.2% and 95.7% in the direct-to-vial group. Also, no differences were found for negative predictive value (82.1, 86.8% and 80.0% respectively).

**Conclusions:**

PapSpin showed good qualitative results in both study arms, even after the material splitting in the first study arm, and is a good alternative to the conventional Pap smear. Additionally, the PapSpin method offers several advantages such as the opportunity to prepare duplicate slides, option for HPV DNA testing and cell block preparations from residual material. Microscopic evaluation of thinner cell preparations is less time consuming than the conventional Pap smears.

## INTRODUCTION

Cervical cancer is still an important health problem. Worldwide, cervical cancer incidence is over 471,000 with mortality rate averaging 230,000 deaths per year is second only to breast cancer.[1] Papanicolaou smears detect preinvasive and early invasive disease, and have led to significant reductions in its incidence and associated mortality in many countries.[2]

The screening Pap test remained unchanged for over half a century, until recently, when several new advances were developed. In 1996, the US Food and Drug Administration (FDA) approved ThinPrep (Hologic, Marlborough MA) liquid-based cytology (LBC) an alternative to the conventional smear. This was followed three years later by the approval of the AutoCyte Prep (now known as SurePath (BD TriPath, Burlington NC). These LBC systems are now well established for cervical cancer screening.[3]

Ample research on ThinPrep and SurePath techniques has evaluated the efficiency of these cytological methods and their technical and economic impact on cytology laboratories and cervical cancer screening.[4] However, there is a lack of studies evaluating the screening efficacy of direct-to-vial systems based on cytocentrifugation. In Central Europe, cytocentrifugation LBC techniques consist of the PapSpin system (ThermoShandon Inc, Pittsburgh, USA), Turbitec (Labonord, Templemars, France) and CytoSCREEN (Seroa, Monaco, Monaco).

Our study was designed to evaluate the diagnostic ability of the PapSpin gynecological cytology system for detecting premalignant cervical lesions. We used a split-sample and direct-to-vial design because there are no direct-to-vial studies evaluating the PapSpin system. The main goal of our study was to compare the qualitative results and diagnostic concordance of concurrently obtained conventional Pap smear and PapSpin preparations, with histology follow-up in the split-sample group. In the direct-to-vial PapSpin group we correlated histological results with abnormal PapSpin test results and determined the sensitivity and specificity of the test.

## MATERIALS AND METHODS

A total of 2,945 women were enrolled during the study period from 2006 to 2008. The screening population consisted of non-pregnant, 30 to 60-year-old women, presenting for routine cervical cancer screening at three large outpatient clinics in the capital city of Vilnius, Lithuania. Gynecologists were trained to collect the samples before the study. Written informed consent was obtained from each patient and comprehensive information was explained to the patient about the tests, including possible risks and the advantages. The protocol for the study, informed consent/agreement form, and the form for the split-sample design with PapSpin microscopic evaluation were approved by the Lithuanian Bioethics Committee of the Ministry of Health Care (2006-02-23, No. 7).

Routine history was recorded and physical exams were performed. All slides and vials were labeled with appropriate patient identification data. In the split-sample group (n = 1,500 women), a cytologic sample was obtained according to a standard procedure using the Papette (Wallach, Orange, CT) brush and a conventional smear was prepared. Subsequently, the Wallach Papette white tip with residual material was removed from its handle and placed into a vial of PapSpin Collection Fluid preservative. In the direct-to-vial group (n = 1,445 women) the Wallach Papette white tip with the cervical sample was removed from its handle and immediately placed into a PapSpin Collection Fluid vial. A conventional smear was not prepared. All specimens were identified with patient study number.

All conventional smears and PapSpin vials were sent to the National Centre of Pathology for processing and for cytology screening. Routine quality control procedures (including the rescreening of 10% negative cases) were followed. In the split-sample group, the conventional Pap smear was processed routinely and separately from PapSpin vials. A study number was given to the PapSpin vial. CytoCheck densitometer was used to monitor the cell density. Standardized cell dilution using Shandon PapSpin preservative fluid was performed according to the manufacturers protocol [Table 1]. The displayed amount of cell suspension was pipetted into the labeled Shandon EZ megafunnel assembly. Using the Cytospin cytocentrifuge, cells from the megafunnel were deposited by cytocentrifugation on to a 22 ×14.75 mm screening area of a glass slide. The slides were fixed and stained with Papanicolaou stain. PapSpin slides were screened blindly and independently from the conventional smears by cytotechnologists and by one pathologist. The Bethesda 2001 system terminology was used for reporting cervical cytology results: NILM (negative for intraepithelial lesion or malignancy), ASC-US (atypical squamous cells of undetermined significance), ASC-H (atypical squamous cell, cannot exclude high grade squamous intraepithelial lesion), LSIL (low grade squamous intraepithelial lesion), HSIL (high grade squamous intraepithelial lesion), SCC (squamous cell carcinoma), AIS (adenocarcinoma *in situ*). Unsatisfactory slides were categorized according to the Bethesda system recommendations for specimen adequacy.

**Table 1:** Standardized cell dilution

*Cell concentration*	*Vol. of cytological material (ml)*	*Vol. of collection fluid (ml)*
Too high cell density	0.2	1.8
High cell density	0.2	1.8
0.2 ml	0.2	1.8
0.3 ml	0.3	1.7
0.4 ml	0.4	1.6
0.5 ml	0.5	1.5
0.6 ml	0.6	1.4
0.7 ml	0.7	1.3
0.8 ml	0.8	1.2
0.9 ml	0.9	1.1
1.0 ml	1	1
1.1 ml	1.1	0.9
1.2 ml	1.2	0.8
1.3 ml	1.3	0.7
1.4 ml	1.4	0.6
1.5 ml	1.5	0.5
1.6 ml	1.6	0.4
1.7 ml	1.7	0.3
1.8 ml	1.8	0.2
1.9 ml	1.9	0.1
2 ml	2	-
3 ml	2	-
4 ml	2	-
5 ml	2	-
“Low cell density”	2	-

For the split sample group, all PapSpin results were compared with the results of the corresponding conventional Pap smear. If more severe lesions were detected on PapSpin, these results were reported to the gynecologist for appropriate clinical management.

Cervical histology (cervical biopsy or loop electrosurgical excision (LEEP) or hysterectomy specimen) was sent to the laboratory for routine processing and interpretation. A histological diagnosis was reported according to the 2003 WHO Tumours of the Breast and Female Genital Organs Classification system: benign, CIN1 (low-grade cervical intraepithelial neoplasia), CIN2 (moderate cervical intraepithelial neoplasia), CIN3/CIS (high-grade cervical intraepithelial neoplasia/ carcinoma *in situ*), SCC (squamous cell carcinoma).

## STATISTICAL ANALYSIS

Diagnostic agreement with pair-matched analyses and kappa-measure were calculated between the two methods (Conventional and PapSpin). Kappa measure was calculated under a dichotomous (positive or negative) classification. Diagnostic sensitivity and specificity with positive and negative predictive values were calculated using CIN2 and above (CIN 2+) as the reference standard for cytological diagnosis of HSIL. A p-value less than 0.05 was considered statistically significant.

## RESULTS

A total of 2,945 women were examined during the study. All the women were grouped into six age groups with five year intervals: 30-34 in group I, 35-39 in group II, 40-44 in group III, 45-49 in group IV, 50-54 in group V, and 55-60 in group VI [Table 2]. The mean age of women was 42.4 years, the youngest was 30 and the oldest was 60 years old (SD ± 9.0). The youngest women (Group I)accounted for 25.9% of the samples and the oldest women (Group VI) for the only 10.5%. There were no differences in the age distribution between study group.

**Table 2:** Distribution of screened women according to their age

Age group	Age	No. of screened women	%
I	30–34	762	25.9
II	35–39	485	16.5
III	40–44	441	15
IV	45–49	533	18.1
V	50–54	415	14.1
VI	55–60	309	10.5

The study found 189 (6.4%) women with abnormal cytology results. Cytological abnormalities were more often detected in 30-39 years old women (42.4%). Abnormalities were detected in 104 (6.8%) cases in the split-sample group and in 85 (5.9%) cases in the direct-to-vial group.

The cytologic diagnosis according to the Bethesda system categories in both study arms is shown in Table 3. In the split-sample group, 31(2.1%) cases were interpreted as ASC-US by the conventional method as compared to 10 (0.7%) by PapSpin (*P* = 0.001). There was no difference in HSIL+ interpretations: 28 (1.9%) coventional *vs* 33 (2.2%) PapSpin (*P* = 0.33). Increased number of LSILs were observed with the PapSpin: 29 (1.9%) *vs* 20 (1.3%) by conventional method. However, this difference was not significant (*P*=0.12). Additionally, a higher proportion of unsatisfactory samples was reported in the conventional smear group: 25 (1.7%) *vs* 6 (0.4%) cases (*P* = 0.001) with the PapSpin. The main advantages of PapSpin test 10.5over conventional smears were better cell preservation with improved cell visualization, reducing the ASC-US and unsatisfactory rates. The microscopic evaluation of thinner cell preparations is less time consuming than conventional Pap smear. The agreement of conventional and PapSpin split samples is shown in Table 4. Kappa measure of agreement showed a good correlation: 0.87 (95% CI: 0.81-0.92).

**Table 3:** Cytological findings in both study groups

*Bethesda category*	*Phase I (n = 1,500)*		*Phase II (n = 1,445)*	
			
	*Conventional smear*	*PapSpin*	*PapSpin only*	
			
	*(% of Total)*	*(% of Total)*	*(% of Total)*
NILM	1387 (92. 5)	1416 (94.5)	1352 (94.5)
Unsatisfactory	25 (1.7)	6 (0.4)	8 (0.6)	
ASC-US	31 (2.1)	10 (0.7)	20 (1.4)
ASC-H	4 (0.3)	3 (0.2)	10 (0.7)	
LSIL	20 (1.3)	29 (1.9)	22 (1.5)
HSIL	28 (1.9)	33 (2.2)	31 (2.1)
AGC	3 (0.2)	1 (0.1)	2 (0.1)
AIS	1 (0.1)	-	-
Squamous	1 (0.1)	1 (0.1)	-
carcinoma			
Metastatic	-	1 (0.1)	-
gastric			
carcinoma			
Total	1,500	1,500	1,445

**Table 4:** Agreement of diagnoses for the PapSpin and conventional cytology in split-sample group

*PapSpin*	*Conventional smear*
	
	*Unsat.*	*NILM*	*ASC-US*	*ASC-H*	*LSIL*	*HSIL*	*AGC*	*AIS*	*SCC*	*PapSpin Totals*
Unsatisfactory	6	-	-	-	-	-	-	-	-	6
WNL	18	1371	20	1	1	2	2	1	-	1416
ASC-US	1	7	2	-	-	-	-	-	-	10
ASC-H	-	1	1	1	-	-	-	-	-	3
LSIL	-	4	7	-	18	-	-	-	-	29
HSIL		2	1	2	1	26	1	-	-	33
AGC	-	1	-	-	-	-	-	-	-	1
Metastatic gastric carcinoma	-	1	-	-	-	-	-	-	-	1
SCC	-	-	-	-	-	-	-	-	1	1
Conventional Totals	25	1387	31	4	20	28	3	1	1	1500

The percent agreement of all diagnoses was 95%

In the direct-to-vial group, 31 (2.1%) HSIL+ and 22 (1.5%) LSIL cases were detected. The frequency of ASC-US was 1.4% and ASC-H-0.7%. The number of unsatisfactory samples was eight cases (0.6%). We did not observe a statisticaly significant difference in HSIL+ detection between split-sample and the direct-to-vial groups (*P* < 0.05).

A total of 141 histological evaluations were performed during the study period. These included 80 (34 LEEP, 36 cervical biopsies, 10 hysterectomies) obtained in the split-sample group and 61 (23 LEEP, 29 biopsy and 9 hysterectomies) in the direct-to-vial group. The indication for surgical management was abnormal cervical cytology (94 cases), endometriosis of the cervix (1), leiomyoma of the uterus (6), endometrial carcinoma (3), borderline serous cystadenoma of the ovary (1), dysgerminoma (1), ovarian adenocarcinoma (2), abnormal colposcopy (15), unknown indications (17).

A comparison of cytological and histological diagnoses in both study groups is presented in Tables 5 and 6. In the split-sample group the sensitivity of conventional and PapSpin tests was 68.7% *vs* 78.1% and the specificity was 93.8% *vs* 91.8% respectively. In the direct-to-vial group, the PapSpin sensitivity was 75.9% and specificity 96.5%. The differences in sensitivity and specificity were not significant.

**Table 5:** Comparison of cytological conventional and PapSpin diagnosis with histological follow-up (split-sample group)

*Histological diagnosis*	*Conventional smear*
	
	*NILM*	*ASC-US*	*ASC-H*	*LSIL*	*HSIL*	*AIS*	*SCC*
Benign	26	5	1	2	1		
CIN1		4		8	2		
CIN2				2			
CIN3/CIS	1	2	3		20	1	
Malignant tumor	2						1

Malignant tumor

	*NILM*	*ASC-US*	*ASC-H*	*LSIL*	*HSIL*	*Carcinoma*	*SCC*

Benign	29	2		3	1		
CIN1	2	1		8	3		
CIN2				2			
CIN3/CIS	2		2		23		
Malignant tumor			1			1	1

**Table 6:** Histological follow up in direct-to-vial group (with PapSpin)

*Histological diagnosis*	*PapSpin*
	
	*NILM*	*ASC-US*	*ASC-H*	*LSIL*	*HSIL*	*Carcinoma*	*Unsatisfactory*
Benign	15	1	2	5	1		1
CIN1			1	4			
CIN2		1	1		5		
CIN3/CIS		1	3	1	16		
Carcinoma					1		1

The positive predictive values for the conventional and PapSpin methods were not different in the split-sample group: 88.0% and 86.2%, but was higher with the direct-to-vial group-95.7%. Also, no differences were found for the negative predictive value: 82.1%, 86.8% and 80.0%, respectively.

**Figure 1:**
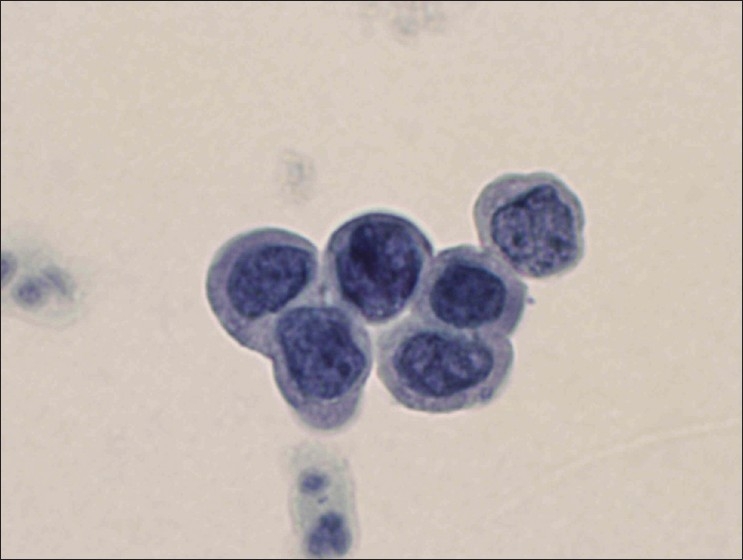
Dysplastic HSIL cells are present on this PapSpin preparation (Papanicolaou stain; original magnification, × 600).

**Figure 2:**
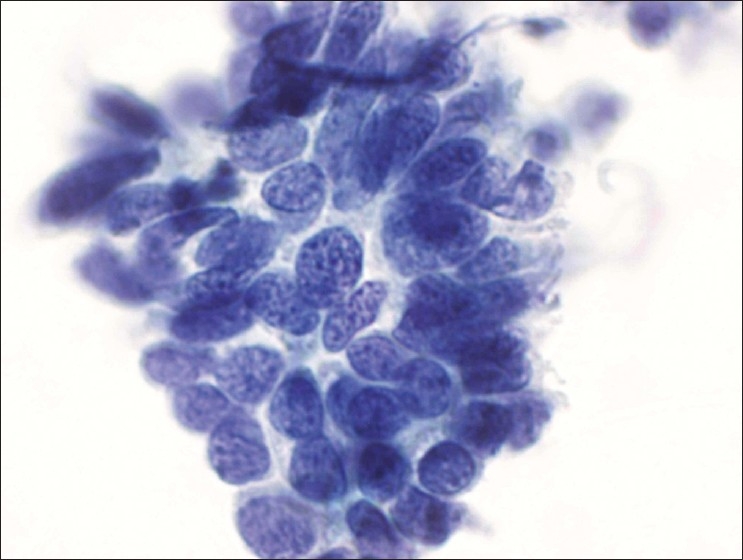
HSIL involving endocervical gland. Tissue fragment. (Papanicolaou stain, original magnification, × 600).

PapSpin cytology preparations in comparison to conventional protocols have optimal preparation quality. Homogenized cytology material was uniformly transferred. The background features including microorganizms were preserved with exception of blood elements. Dysplastic cells [Figure 1] and tissue fragments [Figure 2] were better preserved with PapSpin and the microscopic evaluation of thinner cell preparations of PapSpin was less tiring in comparison to conventional Pap smear.

## DISCUSSION

The current high cost of the widely used ThinPrep and SurePath commercial systems for liquid-based cytology preparations encouraged us to evaluate a less expensive alternative cytopreparatory technique. Cytocentrifugation as PapSpin was considered due to the fact that the Shandon Cytospin (ThermoElectron, Pittsburgh, PA) has been a routine component of most cytology laboratories for 30 years. More flexible regulations for cervical cancer screening tests in Eastern Europe allowed us to evaluate PapSpin technology for cervical screening purposes.

In our study, we evaluated the efficiency of the PapSpin technique for cervical cancer screening in two different sampling settings: the split-sample design, where PapSpin technologies were potentially influenced by splitting the material, and direct-to-vial design, where the whole cervical material was tested with the PapSpin technique. As a reference standard for cytology, we used results on cervical biopsy, LEEP, and hysterectomy specimens.

Split-sample studies of the PapSpin systems effectiveness by Khalbuss *et al*., Weynand *et al*., Garbar *et al*. and Rosenthal *et al*. have already been published in the literature.[5–8] The data from the other studies corresponds with our experience. The comparable rate of epithelial abnormalities were reported by two methods by Weynand *et al*.[6]

One of the advantages of LBC is the low unsatisfactory rate due to optimal cell fixation and easy monitoring of preparation quality. Additionally, this approach allows reprocessing of unsatisfactory samples. Although reprocessing is associated with increased laboratory costs (reagents and labor), it increases the satisfactory rate. Our study’s unsatisfactory rate as compared to the previous studies, are lower for LBC as compared to conventional preparation (0.4% *vs* 1.7%). Satisfactory results were obtained after the reprocessing in 2 out of 6 PapSpin cases in the split-sample group and in 3 out of 8 cases in the direct-to-vial group. The remaining samples had insufficient cytologic material. Because our baseline for conventional and PapSpin unsatisfactory rate was already low, the benefit of improving the unsatisfactory rate was limited due to the participation by the primary health care centers with expierenced physicians.

Currently, the gynecological practice is faced with challenges related to the equivocal ASC-US interpretation. HPV reflex testing, colposcopy, and biopsy are recommended to manage patients with ASC-US cytology.[9] Since its introduction in 1988, ASC-US has been a problematic and controversial diagnosis to define. ASC-US rate is usually monitored in the laboratory to avoid overuse. ASC-US may be due to Pap smear quality (air-dry, crushing artefacts, scant material, thick and bloody smear), sampling, screening, and interpretation. Our study observed lower ASC-US rate of 0.7% for PapSpin in comparison to 2.1% with conventional preparations, primarily due to better cell preservation. In the direct-to-vial group, PapSpin again showed lower frequency of ASC-US than with split-sample conventional 1.2 *vs* 2.1% . Data from The College of American Pathologists PAP program survey indicates a wide range (0.9% to 11%) of ASC-US reporting with ThinPrep and SurePath methods.[10]

In our study, HSIL+ detection rate was higher than that reported by the College of American Pathologists PAP surveys (from 0.1 to 2.0%).[10] This is characteristic for our Lithuanian cervical cancer screening program. According to the data from the Regional State Patient Funds Information System, HSIL+ detection rates vary from 0.2 to 2.2%.

Three HSIL+ cases were missed with conventional cytology in the split-sample test group. Retrospective review revealed that one was missed at the screening level, one was erroneusly categorized due to poor smear quality, and one metastatic gastric tumor was detected in PapSpin preparation with a lack of cells in the conventional test.

We also noticed an increase in LSIL detection with PapSpin in the split sample group. We found 19 (1.3%) with the conventional test *vs* 28 (1.9%) with PapSpin test. Similar observations have been reported by Berenstein *et al*. in their meta-analysis for ThinPrep.[11] These results in our study were associated with improved morphology of koilocyte cytoplasm and better nuclear details.

In the current study, the sensitivity of conventional cytology as compared to PapSpin was 68.7% and 78.1%. Specificity was 93.8% and 91.8% respectively. Split-sample results of our study are similar to those reported by Garbar *et al*.,[7] who reported results with two manual liquid based techniques based on cytocentrifugation. PapSpin test sensitivity of 82.6% and specificity of 96.2% were comparable. Turbitec test also showed similar pattern with sensitivity of 75.0% and specificity of 96. 2%. In our study, PapSpin showed improved sensitivity and specificity for detecting CIN2+ lesions in both the direct-to-vial group and the split sample group. Abulafia et al.[12] concluded that different studies demonstrate a relatively wide range of sensitivity (from about 50 to 90%). To summarize, the PapSpin technique demonstrates efficiency as a cervical cancer screening test and alternative to conventional Pap smear.

## ACKNOWLEDGMENTS

We thank ThermoFischer for provision of 3.000 vials, Pappette brooms, and a cytocentrifuge and gynecologysts Julia Musteikiene (Vilnius Central Outpatients Clinics), Saule Murmiene (Vilnius Regional Central Outpatients Clinics) and Daiva Kersulyte (Karoliniskes outpatients Clinics) as well as her colleagues for collaboartion.

Written consent for publication was obtained from the patient or their relatives.

## COMPETING INTEREST STATEMENT BY ALL AUTHORS

No competing interest to declare by any of the authors.

## AUTHORSHIP STATEMENT BY ALL AUTHORS

All authors of this article declare that we qualify for authorship as defined by ICMJE http://www.icmje.org/#author.

Each author has participated sufficiently in the work and take public responsibility for appropriate portions of the content of this article.

Each author acknowledges that this final version was read and approved.

## ETHICS STATEMENT BY ALL AUTHORS

This study was conducted with approval from Institutional Review Board (IRB) (or its equivalent) of all the institutions associated with this study.

Authors take responsibility to maintain relevant documentation in this respect.

## EDITORIAL/PEER-REVIEW STATEMENT

To ensure integrity and highest quality of CytoJournal publications, the review process of this manuscript was conducted under a double blind model (authors are blinded for reviewers and reviewers are blinded for authors)through automatic online system.

CytoJournal thanks Dr. Bryan Hunt, Cytopathology fellow, Medical College of Wisconsin for the copy editing support during final PDF preparation to improve the final version in collaboration with the authors of the manuscript.Authors with non-english background agreed with the editing support and approved the final version prior to final publication.
